# Collagen type XVIII/endostatin is differentially expressed in primary and metastatic colorectal cancers and ovarian carcinomas

**DOI:** 10.1054/bjoc.2001.2143

**Published:** 2001-11

**Authors:** U Guenther, H Herbst, M Bauer, C Isbert, H-J Buhr, E-O Riecken, D Schuppan

**Affiliations:** Department of Gastroenterology, Surgery, Benjamin Franklin Hospital, Free University, Berlin; Gerhard-Domagk-Institute of Pathology, University of Muenster, Muenster; Department of Gastroenterology and Hepatology, Friedrich-Alexander-University of Erlangen, Erlangen, Germany

**Keywords:** angiogenesis inhibition, cancer, liver, ovary, tumour therapy

## Abstract

Collagen type XVIII (C18) is a nonfibrillar collagen of basement membranes. Its C-terminal fragment, endostatin, has been identified as an inhibitor of angiogenesis. C18 is predominantly expressed by hepatocytes of normal, cirrhotic and neoplastic liver. We compared the patterns of C18 RNA-expression in colonic adenocarcinoma metastases, which represent the most frequently occurring liver tumours, to normal colon mucosa, to primary colon cancers and to ovarian cancers which are often morphologically similar to colonic cancer or metastasis. Two C18-specific RNA-probes were generated to perform in situ hybridization combined with immunohistochemistry for cytokeratin, vimentin and the endothelial marker CD31, in order to characterize the C18-expressing cells. C18/endostatin protein was localized by immunohistology. In colorectal carcinomas and their liver metastases high levels of C18 transcripts were observed in endothelial cells and fibroblasts/myofibroblasts, whereas C18 RNA was virtually absent from carcinoma cells. Ovarian carcinomas displayed high C18 RNA expression both in carcinoma and stromal cells, indicating that induction of C18 transcription in tumour stromal cells is independent of the ability of carcinoma cells to express C18. While the role of tumour cell derived C18 in cancer growth regulation remains unknown, stimulation of proteolysis of the locally strongly expressed C18 to endostatin could offer an attractive approach for a targeted antineoplastic therapy. © 2001 Cancer Research Campaign   http://www.bjcancer.com


*British Journal of Cancer* (2001) **85**(10), 1540–1545
© 2001 Cancer Research Campaign
doi: 10.1054/ bjoc.2001.2143, available online at http://www.idealibrary.com on IDEAL

http://www.bjcancer.com

### Collagen type XVIII/endostatin is differentially expressed in primary and metastatic colorectal cancers and ovarian carcinomas

**U Guenther[1], H Herbst[2], M Bauer[3], C Isbert[4], H-J Buhr[4], E-O Riecken[1] and D Schuppan[3]**

[1]Department of Gastroenterology and [4]Surgery, Benjamin Franklin Hospital, Free University, Berlin; [2]Gerhard-Domagk-Institute of Pathology, University of Muenster, Muenster; and the [3]Department of Gastroenterology and Hepatology, Friedrich-Alexander-University of Erlangen, Erlangen, Germany

**Summary** Collagen type XVIII (C18) is a nonfibrillar collagen of basement membranes. Its C-terminal fragment, endostatin, has been identified as an inhibitor of angiogenesis. C18 is predominantly expressed by hepatocytes of normal, cirrhotic and neoplastic liver. We compared the patterns of C18 RNA-expression in colonic adenocarcinoma metastases, which represent the most frequently occurring liver tumours, to normal colon mucosa, to primary colon cancers and to ovarian cancers which are often morphologically similar to colonic cancer or metastasis. Two C18-specific RNA-probes were generated to perform in situ hybridization combined with immunohistochemistry for cytokeratin, vimentin and the endothelial marker CD31, in order to characterize the C18-expressing cells. C18/endostatin protein was localized by immunohistology. In colorectal carcinomas and their liver metastases high levels of C18 transcripts were observed in endothelial cells and fibroblasts/myofibroblasts, whereas C18 RNA was virtually absent from carcinoma cells. Ovarian carcinomas displayed high C18 RNA expression both in carcinoma and stromal cells, indicating that induction of C18 transcription in tumour stromal cells is independent of the ability of carcinoma cells to express C18. While the role of tumour cell derived C18 in cancer growth regulation remains unknown, stimulation of proteolysis of the locally strongly expressed C18 to endostatin could offer an attractive approach for a targeted antineoplastic therapy. © 2001 Cancer Research Campaign http://www.bjcancer.com

**Keywords**: angiogenesis inhibition; cancer; liver; ovary; tumour therapy

Collagen type XVIII (C18) belongs to a novel class of nonfibrillar collagens forming, together with collagen type XV, the multiplexin (multiple triple-helix domains with interruptions) subgroup within the collagen superfamily. These proteins are characterized by a large globular N-terminal domain, a highly interrupted triple-helical region and a globular C-terminus with four conserved cysteines (Muragaki et al, 1994; Oh et al, 1994a; Rehn and Pihlajaniemi, 1994).

C18 is expressed in highly vascularized organs such as liver, lung, kidney and placenta (Oh et al, 1994b; Muragaki et al, 1995; Rehn and Pihlajaniemi, 1995; Musso et al, 1998; Saarela et al, 1998). Immunohistochemistry suggested a structural role for C18 in the formation of specialized epithelial and endothelial basement membranes (Muragaki et al, 1995; Musso et al, 1998; Saarela et al, 1998). A 22 kDa C-terminal proteolytic fragment of C18, endostatin, has been identified as a potent inhibitor of angiogenesis and tumor growth in mice (Boehm et al, (1997); O'Reilly et al, 1997). Furthermore, endostatin inhibits migration and proliferation and induces apoptosis of endothelial cells in vitro (O'Reilly et al, 1997; Dhanabal et al, 1999a, 1999b; Taddei et al, 1999).

Expression of C18 RNA in normal liver, which was shown to be the major C18-producing organ both in man and rodents (Muragaki et al, 1994, 1995; Oh et al, 1994b; Rehn and Pihlajaniemi, 1995; Musso et al, 1998) is nearly confined to hepatocytes and bile duct epithelia and is up-regulated in these cells in cirrhosis and hepatocellular carcinoma. The high expression by neoplastic hepatocytes in vivo supports a unique role of this collagen not only in the formation of the sinusoidal basement membrane but also in the regulation of angiogenesis and neoplastic transformation (Schuppan et al, 1998).

Here we extended these studies analysing the origin and distribution of C18 in colonic adenocarcinoma metastases as the most frequently occurring secondary liver tumours, compared to normal colonic mucosa and to primary colon cancer. In addition, we studied primary ovarian carcinomas which are difficult to distinguish from colon carcinoma metastases to the ovaries.

*Received 18 April 2001*
*Revised 6 August 2001*
*Accepted 7 August 2001*

*Correspondence to:* D Schuppan, Krankenhausstr. 12, 91054 Erlangen; Tel.: +49-9131-85-33386; Fax: +49-9131-85-36003

### MATERIALS AND METHODS

### Tissues

Tissue samples were obtained from patients undergoing resection for colorectal tumours ($n$ = 16) or partial liver resection for solitary colorectal liver metastases ($n$ = 5). Tissue samples were snap-frozen in liquid nitrogen immediately after removal and submitted for histopathologic diagnosis. The patient data and the histopathologic characteristics are listed in Table 1. As controls, we used colonic tissues with regular histology (at a distance of >10 cm from the focal lesions, $n$ = 6) as well as resectates of ovarian carcinomas ($n$ = 10) and one normal ovary.

### Tissue sectioning and fixation

Frozen sections (7 µm) were collected onto 3-aminopropyl triethoxysilane-coated slides, dried briefly on a hot plate at 80˚C, and fixed

**Table 1** Summary of clinical information and histopathological diagnoses

| Case | Age | Sex | Diagnosis | Histology |
|---|---|---|---|---|
| 1 | 84y | M | sigmoid carcinoma | adenocarcinoma G2, pT4pN1 |
| 2 | 75y | M | rectal carcinoma | adenocarcinoma G2 |
| 3 | 64y | M | colon asc. carcinoma | adenocarcinoma G2, pT3pN1 |
| 4 | 62y | M | rectal carcinoma | adenocarcinoma G2, pT2pN2 |
| 5 | 45y | F | sigmoid carcinoma | adenocarcinoma G3, pT2pN2 |
| 6 | 51y | M | rectal carcinoma | adenocarcinoma G3, pT1pN0 |
| 7 | 75y | M | rectal carcinoma | adenocarcinoma G2, pT3pN0 |
| 8 | 75y | M | rectal carcinoma | mucinous adenocarcinoma G3, pT3pN0 |
| 9 | 68y | M | rectal carcinoma | adenocarcinoma G3, pT3pN2 |
| 10 | 57y | M | rectal carcinoma | adenocarcinoma G2, pT3pN1 |
| 11 | 75y | M | rectal carcinoma | adenocarcinoma G3, pT3pN0 |
| 12 | 82y | M | rectal carcinoma | adenocarcinoma G2, pT3pN0 |
| 13 | 78y | F | rectal carcinoma | adenocarcinoma G2, pT2pN0 |
| 14 | 52y | F | rectal carcinoma | adenocarcinoma G2, pT3pN1 |
| 15 | 65y | F | rectal carcinoma | adenocarcinoma G2, pT2pN0 |
| 16 | 61y | F | sigmoid carcinoma | adenocarcinoma G2, pT4pN0 |
| 17 | 84y | M | sigmoid carcinoma | normal histology distant to lesion |
| 18 (3) | 64y | M | colon asc. carcinoma | normal histology distant to lesion |
| 19 | 79y | F | colon carcinoma | normal histology distant to lesion |
| 20 | 66y | M | colon carcinoma | normal histology distant to lesion |
| 21 | 68y | M | colon carcinoma | normal histology distant to lesion |
| 22 (16) | 61y | F | sigmoid carcinoma | normal histology distant to lesion |
| 23 | 70y | F | colon ca. metastasis | adenocarcinoma G3, pM1HEP |
| 24 | 63y | M | rectal ca. metastasis | adenocarcinoma G2, pM1HEP |
| 25 (21) | 68y | M | colon ca. metastasis | adenocarcinoma G3, pM1HEP |
| 26 (16) | 61y | F | sigmoid ca. metastasis | mucinous adenocarcinoma G3, pM1HEP |
| 27 | 34y | M | colon ca. metastasis | adenocarcinoma G3, pM1HEP |
| 28 | 76y | F | ovarian carcinoma | serous cystadenocarcinoma G3 |
| 29 | 63y | F | ovarian carcinoma | serous cystadenocarcinoma G3 |
| 30 | 86y | F | ovarian carcinoma | serous cystadenocarcinoma G2 |
| 31 | 64y | F | ovarian carcinoma | adenocarcinoma, transitional type G3 |
| 32 | 38y | F | ovarian carcinoma | endometrioid adenocarcinoma G3 |
| 33 | 60y | F | ovarian carcinoma | endometrioid adenocarcinoma G2 |
| 34 | 54y | F | ovarian carcinoma | adenocarcinoma NOS G3 |
| 35 | 76y | F | ovarian carcinoma | serous cystadenocarcinoma G3 |
| 36 | 41y | F | ovarian carcinoma | serous cystadenocarcinoma G2 |
| 37 | 49y | F | ovarian carcinoma | papillary serous cystadenoma, borderline type |
| 38 | 63y | F | ovary | normal histology |

M, male; F, female. Parentheses: number of tissue samples of the same patient.

in 4% paraformaldehyde/phosphate-buffered saline (PBS), pH 7.4, for 20 minutes. After 2 washes in PBS, dehydration in graded ethanols, and short air drying, sections were stored at –80˚C.

### Immunohistology

The immunohistochemical detection of endostatin was performed with polyclonal rabbit antibodies specific for murine endostatin (kind gift of Dr U Eberspaecher and Dr A Menrad, Schering-AG, Berlin, Germany) with cross-reactivity to human endostatin. The alkaline phosphatase anti-alkaline phosphatase (APAAP) method was used for immunohistology, using affinity-purified mouse anti-rabbit immunoglobulin (Dianova, Hamburg, Germany), affinity-purified rabbit anti-mouse immunoglobulin (Dako, Glostrup, Denmark), and the APAAP complex (1:20 dilution, Dako). Alkaline phosphatase was developed with new fuchsin.

### Preparation and labelling of probes

The probes were generated by oligo(dT)-primed reverse transcription of human liver RNA followed by amplification with oligo-deoxyribonucleotide primers corresponding to nucleotides 1483–1501 (CGA CCC ACA AGC CCA CCC G) and 2083–2062 (TCT CCG GCC ATC TGC ATC CAG G) for the endostatin-encoding region, and to nucleotides 168–190 (GGG ACC TGT GGT CTA CGT GTC GG) and 809–787 (TCG CCT TTC TGT CCT GCA TCA CC) for the collagenous domain of C18 (Oh et al, 1994b). The amplicons were cloned into the EcoRV site of pZErO-1 (Invitrogen, Leek, Netherlands) and further characterized by restriction digests and sequence analysis. After appropriate linearization of the plasmids, T7 or SP6 RNA-polymerase (Gibco-BRL, Eggenstein, Germany), respectively, were employed to obtain run-off transcripts of either the anti-sense (complementary to cellular RNA), or sense (control) strands. Transcription and labelling of RNA probes were performed as described using [$^{35}$S]-uridine-5′-(α-thio)-triphosphate (1250 Ci mmole$^{-1}$, New England Nuclear, Dreieich, Germany) (Milani et al, 1990; Herbst et al, 1997). The specific activity routinely obtained was 1.2–1.4 $\times$ $10^9$ cpm µg$^{-1}$.

### In situ hybridization

Pre-hybridization, hybridization, washing procedures including removal of non-specifically bound probe by ribonuclease A digestion and autoradiography of slides were as described before (Milani et al, 1990; Herbst et al, 1997). Controls included hybridization with C18 sense probes as well as with a procollagen α1(I) probe (Milani et al, 1990).

*© 2001 Cancer Research Campaign* 

### Immunohistology combined with in situ hybridization

Sections were stained with monoclonal antibodies specific for cytokeratin (clone KL-1, Dako), vimentin (clone V9, Dako), smooth muscle α-actin (clone 1A4, Sigma Chemical Co, St Louis, MO), and the endothelial cell marker CD31 (clone JC70A, Dako) using the immunoalkaline phosphatase (APAAP) method as described above (Herbst et al, 1997).

### RESULTS

### Normal colon

Some C18 RNA-expressing cells were found within the lamina muscularis propria, particularly in interspersing stromal tissue. Only very few positive cells were seen in the lamina propria. Epithelial cells did not display C18 RNA at detectable levels (Figure 1A).

### Colorectal carcinomas and colorectal liver metastases

A highly increased C18 RNA-expression was observed in peri-tumourous stromal cells, both of primary tumours and metastases. The phenotype of these C18-positive cells was consistent with stromal fibroblasts or endothelial cells of small vessels and capillaries. C18 RNA was only marginally detectable in the carcinoma cells. Only a few tumour cells in 5 of 16 cases of colorectal carcinomas and 2 of 5 cases of colorectal liver metastases displayed a weak signal slightly above the background (Figure 1B–D). In colorectal liver metastases the C18 RNA-expression in the adjacent liver tissue showed a homogeneous labelling of all hepatocytes as previously described (Schuppan et al, 1998). In situ hybridizations performed with both of the C18-specific RNA-probes, encoding either the endostatin-domain or part of the collagenous region, revealed an identical expression pattern.

By combined immunohistochemical staining and in situ hybridization the C18 RNA-expressing cells in the tumour stroma were identified as vimentin-positive mesenchymal cells. Many of the C18 RNA-positive cells also expressed smooth muscle α-actin, a marker of a myofibroblastic differentiation, or the endothelial CD31 antigen (Figure 2A), but cells were consistently negative for cytokeratin (Figure 2B).

By immunostaining, the C18/endostatin protein was found within the tumour stroma, with prominent labelling of endothelial cells of small vessels and capillaries, well in agreement with the results of in situ hybridization. Carcinoma cells did not display endostain immunoreactivity, whereas the sinusoidal basement membranes of neighbouring liver parenchyma showed a linear but weaker deposition of C18/endostatin (Figure 2E, F).

### Ovarian carcinomas

In contrast to colon carcinomas, ovarian carcinoma cells showed highly elevated C18 RNA transcript levels in 8 of 10 cases. However, as in colorectal carcinomas and colorectal liver metastases, C18 mRNA expression was high in the tumour stroma (Figure 1E, F). C18 transcripts were almost homogeneously distributed among the groups of tumour cells without variation along morphologically distinct sites such as the front of invasion vs. more central tumour regions. There was no relationship of C18 transcript levels with cellular or nuclear characteristics of the tumour cells. Double-labelling experiments documented that the vast majority of the cytokeratin-positive carcinoma cells were expressing C18 RNA (Figure 2C). In the 2 cases that displayed a transitional or endometrioid-type morphology only a weak signal for C18 RNA was detected over the carcinoma cells (not shown). As in colon carcinomas, combined immunohistochemical staining and in situ hybridization allowed identification of the C18 RNA-expressing cells in the tumour stroma as vimentin (α-actin)-positive fibroblasts/myofibroblasts or anti-CD31-positive endothelial cells (Figure 2D).

In one case of normal ovary no C18 RNA transcripts were detectable, whereas the neighbouring epithelial lining of the Fallopian tube displayed a weak signal. In situ hybridizations with sense (non-complementary control) probes revealed a weak, nonspecific background labelling (not shown). High procollagen α1(I) transcript levels were detectable over fibroblastic cells of all specimens as proof for integrity of the examined tissue samples (not shown).

### DISCUSSION

As a component of specialized basement membranes, C18 merits particular attention, since its proteolysis by proteases such as cathepsin L and elastase generates the potent angiogenesis inhibitor endostatin (Wen et al, 1999; Felbor et al, 2000). Previous quantitative and in situ hybridization studies revealed a prominent expression of C18 mRNA and protein in epithelia of liver and to a minor degree in kidney and lung (Oh et al, 1994b; Muragaki et al, 1995; Rehn and Pihlajaniemi, 1995; Musso et al, 1998; Schuppan et al, 1998). However, the patterns of C18 RNA expression in primary colonic adenocarcinoma and its metastases as the most frequently occurring secondary liver tumours, compared to normal colon as well as to often morphologically similar ovarian carcinomas is largely unknown.

Expression patterns of C18 in the liver tissue adjacent to the metastases displayed high transcript levels in hepatocytes, and lower levels in epithelial cells of small bile ducts and in endothelial cells as described previously (Schuppan et al, 1998). However, epithelia of colon carcinoma did not express C18 mRNA, while high C18 RNA transcript levels were present in the tumour stroma of both primary neoplasms and liver metastases. Here, fibroblasts and endothelial cells were identified as prominent sources of C18.

In contrast, ovarian carcinomas showed a strong C18 RNA expression in tumour cells in 8 out of 10 cases. Also, C18 expression was highly elevated in tumour endothelial cells and fibroblasts in these tumours. The finding of a high epithelial expression of C18 in ovarian cancer is of practical interest, since ovarian carcinoma may spread to the intestinal wall and may then be present in biopsy materials. Vice versa, gastrointestinal carcinomas may spread to the ovary. However, on microscopic examination ovarian endometrioid and mucinous carcinomas are often indistinguishable from colon colorectal carcinoma metastatic to or infiltrating the ovary, even when using the tumour markers CEA and CA125. Therefore, C18 may serve as a novel marker to distinguish carcinoma of intestinal (absent C18 expression) vs. ovarian (high C18 expression) origin.

Our study demonstrates that epithelial cells of different origin differ widely in their ability to express C18. While the low C18 expression of normal colonic epithelium is largely maintained in its neoplastic counterparts, and a high C18 expression is found both in normal and neoplastic hepatocytes (Schuppan et al, 1998), C18 is clearly up-regulated in the epithelium of ovarian carcinomas. The finding of variable C18 expression in adenocarcinomas depending

*© 2001 Cancer Research Campaign*

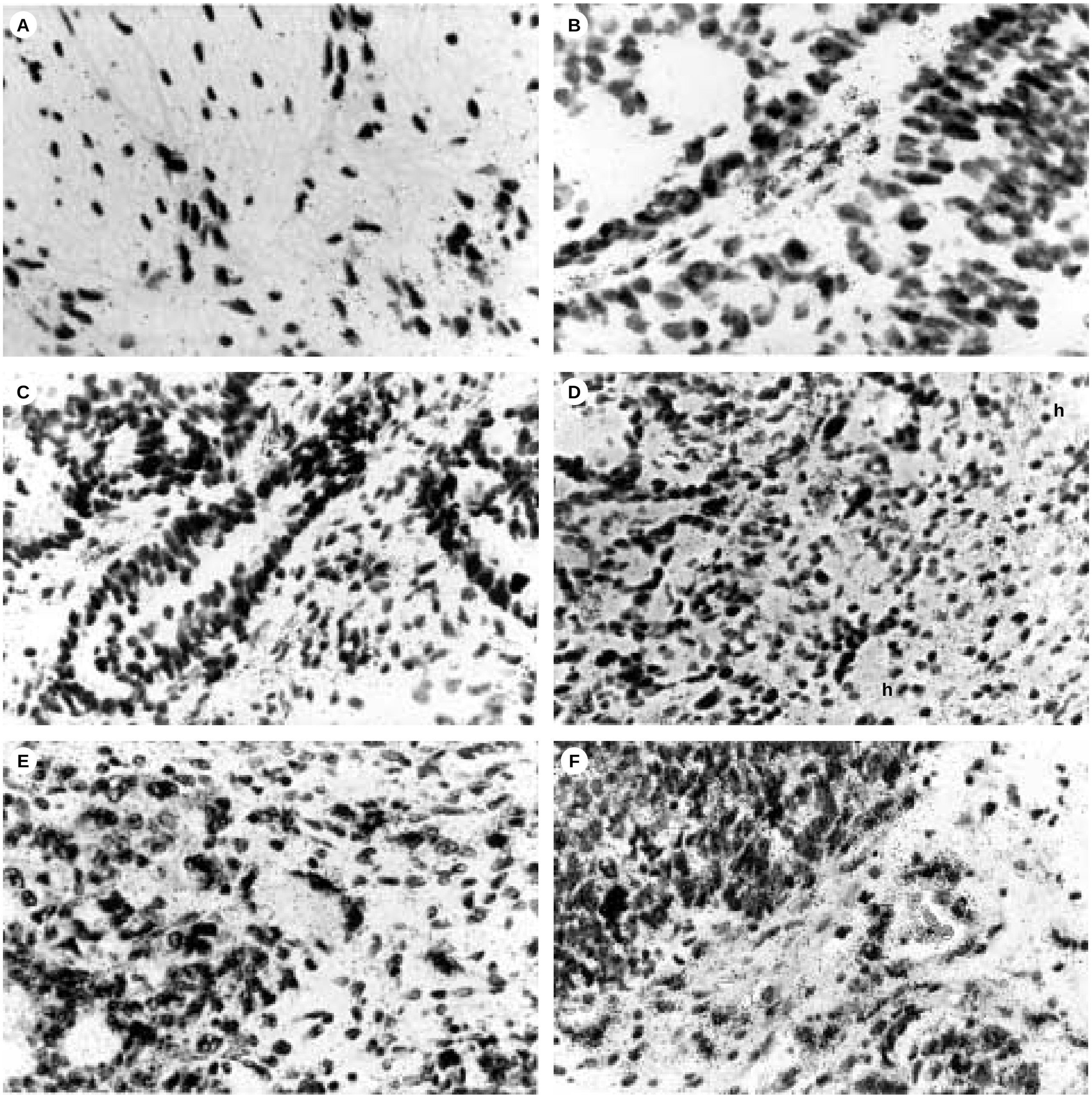

**Figure 1** In situ hybridization with the [$^{35}$S]-labelled antisense RNA-probe for the endostatin-domain of C18 in normal colon (case 17; **A**), colon carcinoma (cases 4, 9; **B**, **C**), colorectal liver metastasis (case 24; **D**) and ovarian carcinoma (cases 32, 34; **E**, **F**). In normal colon some C18 RNA-expressing cells are found within the lamina muscularis propria (**A**). In colorectal carcinomas and colorectal liver metastases a highly upregulated C18 RNA-expression is seen in the tumour stroma. Labelled cells are morphologically consistent with fibroblasts and endothelial cells. Carcinoma cells do not express C18 RNA at detectable levels (**B**–**D**). Homogenous labelling of all hepatocytes (h) is present within the parenchyma adjacent to the liver metastasis (**D**). Tumour cells of ovarian carcinomas display strong and homogeneous C18 RNA-expression, as do fibroblasts and endothelial cells of the tumour stroma (**E**, **F**). Autoradiographic exposure time 35 days. Original magnification × 170 (**A**), × 120 (**B**–**F**)

on their site of origin argues against a pivotal function of C18 in early neoplastic transformation, but does not exclude a role of C18 in progression of ovarian carcinoma. In all cases, however, induction of peritumoural C18 expression by stromal endothelial cells and fibroblasts appears to be relevant for tumour progression and metastatic behaviour.

Whereas intact C18 may promote endothelial cell survival and angiogenesis, its C-terminal proteolytic fragment, endostatin, has been shown to be a potent inhibitor of angiogenesis (Boehm et al, 1997; O'Reilly et al, 1997). Cathepsin L, one of the proteases implicated in proteolytic cleavage of endostatin from its precursor C18, is widely expressed in colorectal carcinomas as well as in the colorectal cancer cell line HT-29 (Sheahan et al, 1989; Chauhan et al, 1991; Shuja et al, 1991; De Stefanis et al, 1997). Thus, epithelial tumours potentially modulate their growth by generating the proteases liberating endostatin from C18. The balance between C18 expression and cathepsin L, elastase or other proteases possibly involved in C18 processing may, therefore, be central for the regulation of angiogenesis and the dynamics of tumour growth. Importantly, the local production of C18 by the tumour cells opens

*© 2001 Cancer Research Campaign*

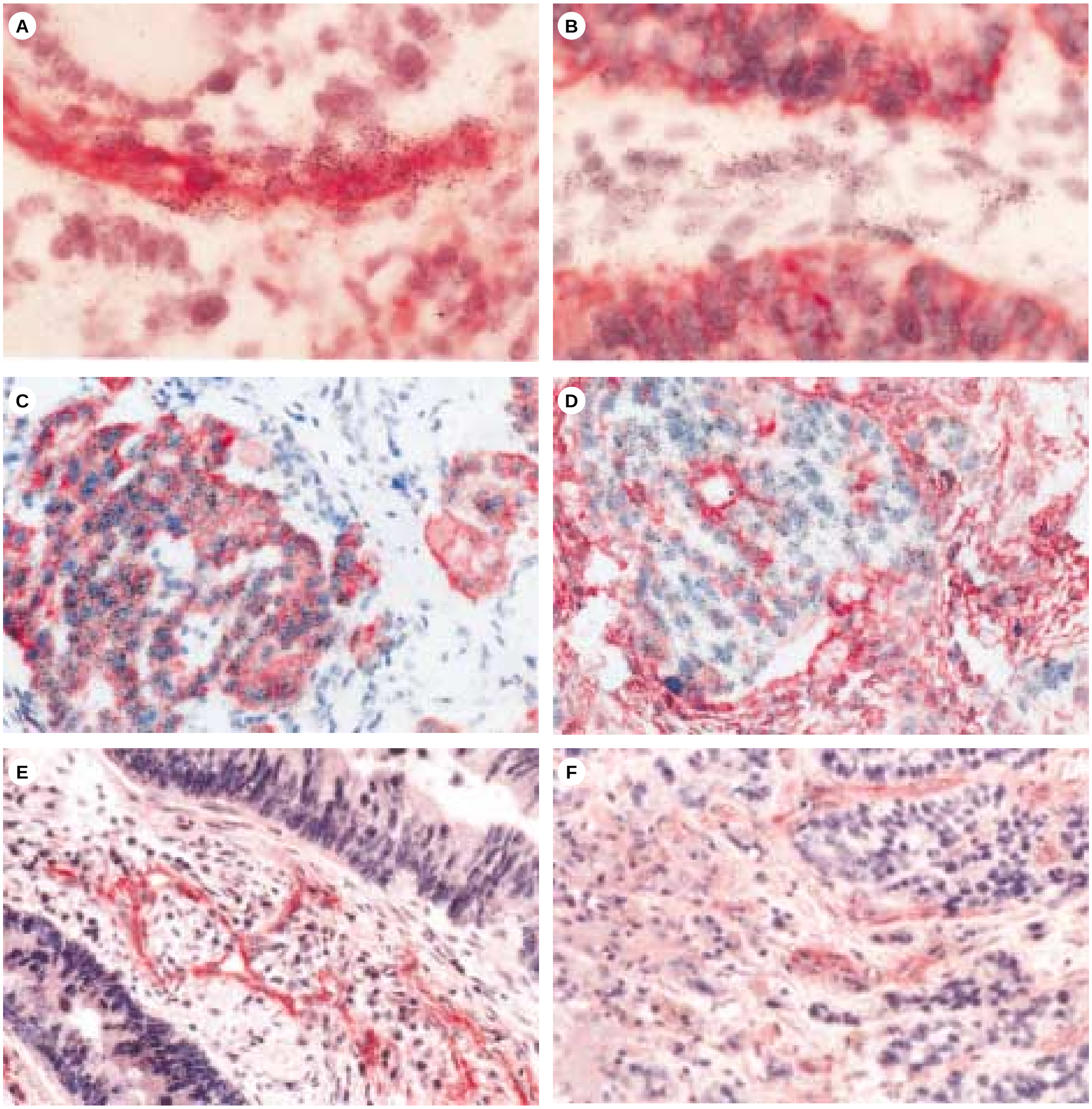

**Figure 2** Immunostaining for the endothelial cell specific marker CD31 (**A**), cytokeratin (characteristic for epithelial cells, **B**, **C**) and vimentin (characteristic for mesenchymal cells, **D**) in a colon carcinoma (case 6; **A**, **B**) and an ovarian carcinoma (case 28; **C**, **D**) followed by in situ hybridization with [$^{35}$S]-labelled C18 antisense RNA-probes for the endostatin domain. In colorectal carcinomas prominent C18 RNA expression is observed in the tumour stroma. Many of the C18-expressing cells are identified as endothelial by their co-expression of CD31 (**A**). Virtually all cytokeratin-positive carcinoma cells lack expression of C18 RNA (**B**). In contrast, ovarian carcinomas display strong C18 RNA-expression in the vast majority of the cytokeratin-positive tumour cells (**C**), as well as in some vimentin-positive mesenchymal cells of the tumour stroma (**D**). Immunostaining for C18/endostatin in a colorectal carcinoma (case 4; **E**) and a colorectal liver metastasis (case 27; **F**) localizes the protein in the tumour stroma, particularly in and around endothelial cells of small vessels and capillaries. Within normal liver tissue adjacent to the colorectal liver metastasis a weaker endostatin staining is found along the perisinusoidal space (open arrows; **F**). Cryostat sections; APAAP procedure; autoradiographic exposure time 56 days (**A**–**D**). Original magnification $\times$ 250 (**A**, **B**), $\times$ 100 (**C**–**F**)

the opportunity to suppress tumour growth via local induction of these proteases. Furthermore, C18 expression may be used to characterize the primary site of ill-defined adenocarcinomas.

### ACKNOWLEDGMENTS

The authors thank Dr U Eberspaecher and Dr A Menrad for their kind gift of endostatin antibodies. Supported by Deutsche Forschungsgemeinschaft (grant He 1330/2–1), the Johann and Frieda Marohn Foundation, the German Cancer Foundation and the Federal Ministry of Science & Research (BMBF-IZKF, Univ. Erlangen-Nuernberg).

### REFERENCES

Boehm T, Folkman J, Browder T and O'Reilly MS (1997) Antiangiogenic therapy of experimental cancer does not induce acquired drug resistance. *Nature* **390**: 404–407

 *© 2001 Cancer Research Campaign*

Chauhan SS, Goldstein LJ and Gottesman MM (1991) Expression of cathepsin L in human tumors. *Cancer Res* **51**: 1478–1481

De Stefanis D, Demoz M, Dragonetti A, Houri J, Ogier-Denis E, Codogno P, Baccino FM and Isidoro C (1997) Differentiation-induced changes in the content, secretion, and subcellular distribution of lysosomal cathepsins in the human colon cancer HT-29 cell line. *Cell Tissue Res* **289**: 109–117

Dhanabal M, Ramachandran R, Volk R, Stillman IE, Lombardo M, Arispe L, Simons M and Sukhatme VP (1999a) Yeast production, mutants, and antitumor effect in renal cell carcinoma. *Cancer Res* **59**: 189–197

Dhanabal M, Ramchandran R, Waterman MJF, Lu H, Knebelmann B, Segal M and Sukhatme VP (1999b) Endostatin induces endothelial cell apoptosis. *J Biol Chem* **274**: 11721–11726

Felbor U, Dreier L, Bryant RAR, Ploegh HL, Olsen BR and Mothes W (2000) Secreted cathepsin L generates endostatin from collagen XVIII. *EMBO J* **19**: 1187–1194

Herbst H, Wege T, Milani S, Grappone G, Orzechowski HD, Bauer M, Bechstein WO, Neuhaus P, Gressner AM and Schuppan D (1997) Tissue inhibitor of metalloproteinase-1 and -2 expression in rat and human liver fibrosis. *Am J Pathol* **150**: 1647–1659

Milani S, Herbst H, Schuppan D, Surrenti C, Riecken EO and Stein H (1990) Cellular localization of type I, III, and IV procollagen gene transcripts in normal and fibrotic human liver. *Am J Pathol* **137**: 59–70

Muragaki Y, Abe N, Ninimiya Y, Olsen BR and Ooshima A (1994) The human alpha 1 (XV) collagen chain contains a large amino-terminal non-triple helical domain with a tandem repeat structure and homology to alpha 1 (XVIII) collagen. *J Biol Chem* **269**: 4042–4046

Muragaki Y, Timmons S, Griffith CM, Oh SP, Fadel B, Quertermous T and Olsen BR (1995) Mouse Col18al is expressed in a tissue-specific manner as three alternative variants and is localized in basement membrane zones. *Proc Natl Acad Sci USA* **92**: 8763–8767

Musso O, Rehn M, Saarela J, Theret N, Lietard J, Hintikka E, Lotrian D, Campion JP, Pihlajaniemi T and Clement B (1998) Collagen XVIII is localized in sinusoids and basement membrane zones and expressed by hepatocytes and activated stellate cells in fibrotic human liver. *Hepatology* **28**: 98–107

Oh SP, Kamagata YM, Muragaki Y, Timmons S and Olsen BR (1994a) Isolation and sequencing of cDNAs for proteins with multiple domains of Gly-Xaa-Yaa repeats identify a distinct family of collagenous proteins. *Proc Natl Acad Sci USA* **91**: 4229–4233

Oh SP, Warman ML, Seldin MF, Cheng SD, Knoll JH, Timmons S and Olsen BR (1994b) Cloning of cDNA and genomic DNA encoding human type XVIII collagen and localization of the alpha 1 (XVIII) collagen gene to mouse chromosome 10 and human chromosome 21. *Genomics* **19**: 494–499

O'Reilly MS, Boehm T, Shing Y, Fukai N, Vasios G, Lane WS, Flynn E, Birkhead JR, Olsen BR and Folkman J (1997) Endostatin: An endogeneous inhibitor of angiogenesis and tumor growth. *Cell* **88**: 277–285

Rehn M and Pihlajaniemi T (1994) Alpha 1 (XVIII), a collagen chain with frequent interruptions in the collagenous sequence, a distinct tissue distribution, and homology with type XV collagen. *Proc Natl Acad Sci USA* **91**: 4234–4238

Rehn M and Pihlajaniemi T (1995) Identification of three N-terminal ends of type XVIII collagen chains and tissue-specific differences in the expression of the corresponding transcripts. *J Biol Chem* **270**: 4705–4711

Saarela J, Rehn M, Oikarinen A, Autio-Harmainen H and Pihlajaniemi T (1998) The short and long forms of type XVIII collagen show clear tissue specificities in their expression and location in basement membrane zones in humans. *Am J Pathol* **153**: 611–626

Schuppan D, Cramer T, Bauer M, Strefeld T, Hahn EG and Herbst H (1998) Hepatocytes as a source of collgen type XVIII endostatin. *Lancet* **352**: 879–880

Sheahan K, Shuja S and Murnane MJ (1989) Cysteine protease activities and tumor development in human colorectal carcinoma. *Cancer Res* **49**: 3809–3814

Shuja S, Sheahan K and Murnane MJ (1991) Cysteine endopeptidase activity levels in normal human tissues, colorectal adenomas and carcinomas. *Int J Cancer* **49**: 341–346

Taddei L, Chiarugi P, Brogelli L, Cirri P, Magnelli L, Raugei G, Ziche M, Granger HJ, Chiarugi V and Ramponi G (1999) Inhibitory effect of full-length human endostatin on in vitro angiogenesis. *Biochem Biophys Res Commun* **263**: 340–345

Wen W, Moses MA, Wiederschain D, Arbiser JL and Folkman J (1999) The generation of endostatin is mediated by elastase. *Cancer Res* **59**: 6052–6056

*© 2001 Cancer Research Campaign*
